# Atypical Presentation of Degenerative Cervical Myelopathy: A Case Report

**DOI:** 10.26502/acmcr.96550760

**Published:** 2026-06-24

**Authors:** Sumanjali Reddy Kanmantha Reddy, Amirpasha Ehsan, Kiin Kim, Marcel P. Fraix, Devendra K. Agrawal

**Affiliations:** 1 Department of Translational Research, College of Osteopathic Medicine of the Pacific, Western University of Health Sciences, Pomona, CA 91766, USA; 2 Providence St. Jude Medical Center, Fullerton, CA 92835, USA; 3 Department of Physical Medicine and Rehabilitation, College of Osteopathic Medicine of the Pacific, Western University of Health Sciences, Pomona, CA 91766 USA

**Keywords:** Cervical cord pathology, Degenerative cervical myopathy, Degenerative disease, Laminectomy, Lumbar spinal stenosis, Neurogenic claudication, Neurologic deficit, Ossification of the posterior longitudinal ligament (OPLL), Paresthesia, Spinal cord dysfunction

## Abstract

Degenerative cervical myelopathy (DCM) is a progressive condition most caused by degenerative changes that lead to spinal canal stenosis and characteristic neurologic deficits involving both upper and lower extremities. We report an atypical presentation of DCM manifesting exclusively as new-onset lower extremity weakness following lumbar decompression surgery, without neck pain or upper extremity symptoms. This case highlights the importance of maintaining a high index of suspicion for cervical myelopathy and performing whole-spine imaging in patients with unexplained postoperative neurologic deficits.

## 1. Introduction

Degenerative cervical myelopathy (DCM) is the most common cause of nontraumatic spinal cord dysfunction in adults worldwide. It results from progressive degenerative changes such as disc herniation, ligamentous hypertrophy or ossification, and osteophyte formation, leading to spinal canal narrowing and cord compression. Typical clinical manifestations include neck pain, upper extremity weakness or sensory deficits, impaired hand dexterity, gait disturbance, and pathologic reflexes [1, 2]. Isolated lower extremity symptoms without accompanying cervical or upper extremity findings are uncommon and may represent a false localizing sign, increasing the risk of delayed diagnosis. We present a case of cervical cord compression discovered only after lumbar surgery, emphasizing the importance of imaging the entire spine in patients with unexplained postoperative neurologic deficits.

## 2. Case Presentation

A 67-year-old male with a history of chronic low back pain secondary to lumbar spinal stenosis with neurogenic claudication, lumbar radiculopathy, meralgia paresthetica, hypertension, and hyperlipidemia presented for elective L1–L4 laminectomies and foraminotomies. He reported a two-year history of lower back pain and right anterior thigh pain exacerbated by standing and walking and relieved by recumbency. He denied bowel or bladder dysfunction, saddle anesthesia, or lower extremity weakness.

Preoperative examination demonstrated a positive straight leg raise with paresthesia in a bilateral L5/S1 distribution. Lumbar MRI revealed severe multilevel degenerative changes with significant canal stenosis at L1–L4. Conservative management, including physical therapy, medications, and epidural steroid injections, failed to provide sustained relief, prompting surgical intervention. The patient underwent uncomplicated L1–L4 decompression. On postoperative days (POD) 0 and 1, he reported improvement in preoperative pain and denied weakness or sensory deficits. On POD 3, he developed new bilateral proximal lower extremity weakness accompanied by numbness and paresthesia in the plantar aspects of both feet. He denied back pain, radicular symptoms, or upper extremity complaints.

Neurologic examination demonstrated preserved upper extremity strength, intact reflexes, and absence of Hoffman’s sign or clonus. Laboratory studies, including creatine kinase, were normal. MRI of the lumbar and thoracic spine showed adequate decompression without cord or conus injury. Given persistent lower extremity weakness without lumbar pathology, neurology recommended cervical spine imaging.

**Figure 1:**
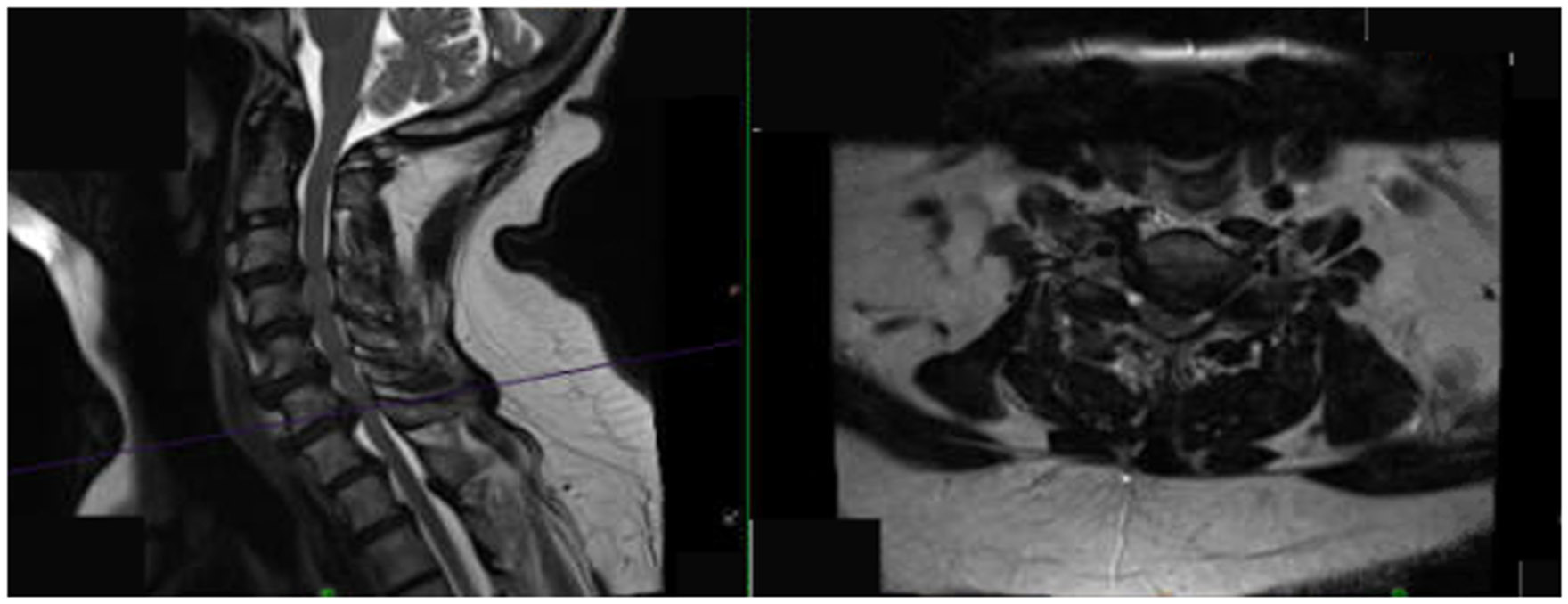
MRI of cervical spine demonstrating levels of degenerative disc disease and levels of vertebral canal stenosis secondary to ossification of the posterior longitudinal ligament at the vertebral canal, most significant at C6-7.

**Figure 2:**
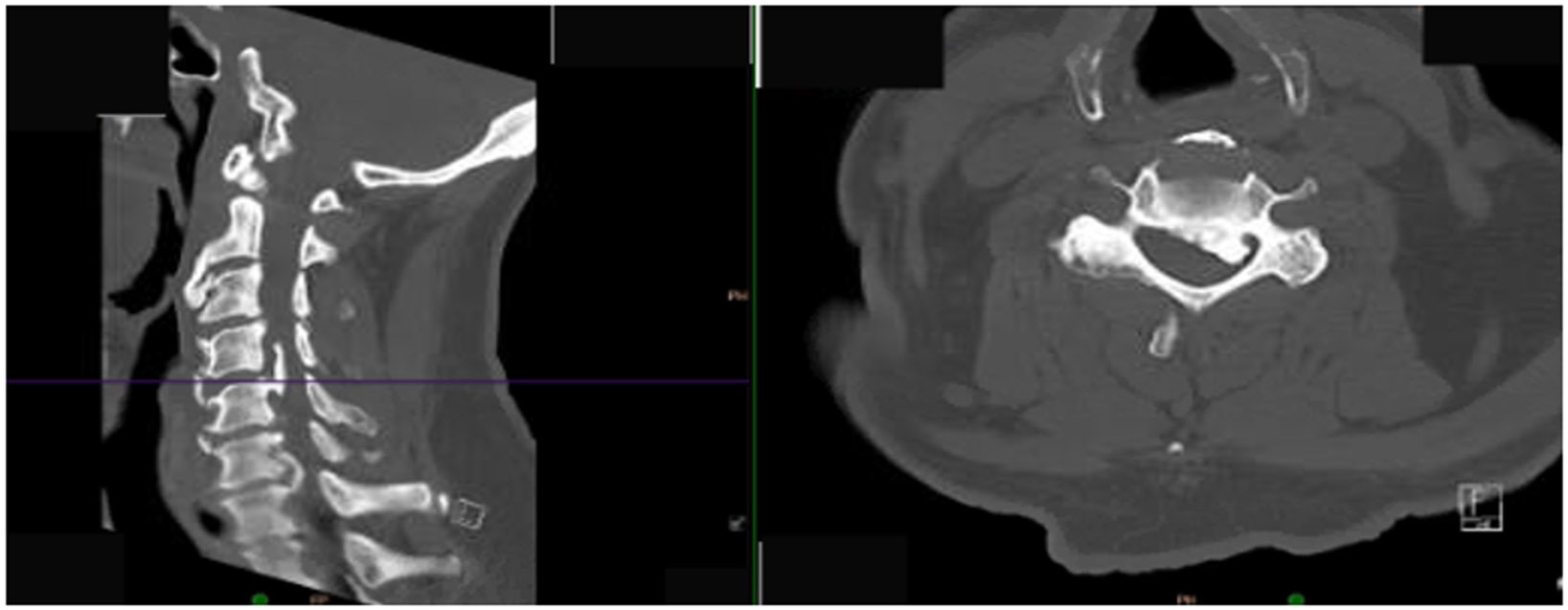
CT of cervical spine demonstrating severe stenosis with clear evidence of impingement of cord at C4-5 and C6-7 with prominent ossification of posterior longitudinal ligament and calcification.

MRI of the cervical spine revealed severe multilevel spinal canal stenosis at C4–5 and C6–7 with cord indentation with findings clinically consistent with cervical myelopathy from spinal cord compression (Figure 1). CT imaging demonstrated ossification of the posterior longitudinal ligament (OPLL), most pronounced at C6–7 (Figure 2). The patient subsequently underwent C3–C7 posterior decompression and fusion after a brief delay to allow resolution of suspected cord edema. Postoperatively, the patient experienced significant improvement in lower extremity strength and sensory symptoms. At outpatient follow-up, he reported complete resolution of neurologic deficits.

## Discussion

Degenerative cervical myelopathy has an estimated prevalence of 605 per million in North America and remains a leading cause of spinal cord dysfunction in older adults [3]. Although classically associated with upper extremity symptoms and gait disturbance, DCM may present atypically, including isolated lower extremity pain or weakness due to false localizing signs [4, 5]. Lower extremity symptoms resulting from cervical cord compression are thought to represent “tract pain,” caused by irritation or deformation of ascending spinothalamic pathways within the spinal cord [6]. Several case reports have described patients with lower limb sensory disturbances or sciatica-like pain who were initially treated for lumbar pathology before cervical compression was identified [6, 7].

In this case, the patient’s new postoperative lower extremity weakness was initially concerning for lumbar surgical complications. However, thorough evaluation and whole-spine imaging revealed severe cervical stenosis with OPLL, ultimately responsible for his symptoms. The absence of neck pain, upper extremity weakness, or pathologic reflexes contributed to diagnostic delay. This case underscores the importance of considering cervical cord pathology in patients with unexplained lower extremity neurologic deficits, particularly when symptoms are discordant with imaging findings at the presumed level of pathology.

## Conclusions

New or unexplained lower extremity weakness following lumbar spine surgery should prompt evaluation of the entire neuraxis (brain and spine). DCM may present without classical symptoms and can manifest as a false localizing sign affecting the lower extremities. Early recognition through comprehensive spinal imaging is essential to prevent diagnostic delay and optimize neurologic recovery.
